# Serum uric acid to serum creatinine ratio predicts neurological deterioration in branch atheromatous disease

**DOI:** 10.3389/fneur.2023.1098141

**Published:** 2023-01-20

**Authors:** Yinglin Liu, Honglei Wang, Ronghua Xu, Lanying He, Kun Wu, Yao Xu, Jian Wang, Fan Xu

**Affiliations:** ^1^Department of Neurology, Chengdu Second People's Hospital, Chengdu, Sichuan, China; ^2^Department of Neurology, Yibin Second People's Hospital, Yibin, Sichuan, China; ^3^Department of Laboratory, Yibin Sixth People's Hospital, Yibin, Sichuan, China; ^4^Department of Radiology, Pingshan County People's Hospital, Chengdu, Sichuan, China; ^5^Department of Public Health, School of Public Health, Chengdu Medical College, Chengdu, Sichuan, China

**Keywords:** branch atheromatous disease, early neurological deterioration, SUA/SCr, uric acid, prognosis

## Abstract

**Background and objective:**

Branch atheromatous disease (BAD) makes patients prone to early neurological deterioration (END), resulting in poor prognosis. The aim of this study was to investigate the association between SUA/SCr and END in BAD stroke patients.

**Methods:**

We conducted a retrospective study that included 241 patients with BAD-stroke within 48 h of symptom onset. We divided the patients into the END group and the no END group. END was defined as an NIHSS score increase of more than 2 points within 1 week. SUA/SCr was calculated by the concentration of serum uric acid and creatine (serum uric acid/serum creatine) on admission. Univariate and multivariate analyses were used to identify independent predictors of END in BAD-stroke patients.

**Results:**

END was observed in 24.1% (58/241) of the patients in our study. Multiple logistic regression analyses showed that SUA/SCr (aOR, 0.716; 95% CI, 0.538–0.952; *P* = 0.022) and female sex (aOR, 0.469; 95% CI, 0.245–0.898; *P* = 0.022) were associated with END after adjusting for confounding factors. The predicted value of SUA/Scr for END was a sensitivity of 79.3%, a specificity of 44.8%, and an AUC of 0.609 (95% CI, 0.527–0.691, *P* < 0.05). The optimal cut-off value was 4.76.

**Conclusion:**

SUA/SCr was negatively associated with the risk of END in BAD stroke patients.

## 1. Introduction

Branch atheromatous disease (BAD), which was initially put forward by Caplan in 1989, is a specific type of stroke caused by atheromatous occlusion at the orifice of large caliber penetrating arteries, is characterized by special MRI manifestations and makes patients prone to neurological deterioration in the early phase (1–4). Although the definition of BAD has not been fully set up yet, it is universally accepted that BAD is a single subcortical infarction and lack of severe stenosis of the parent artery that supplies the regions of deep perforators (5). The study indicated that BAD might show a larger lesion size and a greater tendency of neurologic worsening than lacunar infarction, although both disorders are forms of intracranial deep brain infarction (6). One large study found that the incidence of BAD in ischaemic stroke was 9.74%; however, the incidence of END was as high as 39.4% (7). Previous studies have shown that early neurological deterioration (END) in BAD patients is associated with various factors, such as infarct size, infarct location, female sex, severe neurological deficit, and platelet parameters (4, 8–12). Given the strong association between END and long-term clinical outcome, the END had been the most attentive clinical problem in BAD. So it is significant to assess the risk of END. Serum uric acid (SUA) was reported to be associated with the development and prognosis of cerebrovascular disease (13–17). Some studies showed that hypouricaemia was related to reduced neurological deterioration, improved outcome and lower in-hospital mortality in patients with cerebral infarction (13, 15, 18). A tertiary analysis of the URICO-ICTUS trial suggested that uric acid therapy significantly reduced the incidence of early ischaemic worsening compared with placebo in patients treated with alteplase within 4.5 h of onset (13). Some studies have indicated that hyperuricaemia is a significant protective factor in ischaemic stroke (19, 20). In addition, a recent meta-analysis showed that there was no significant correlation between SUA levels and the prognosis of ischaemic stroke (21). At present, the conclusion remains controversial. Studies have also suggested that the effect of SUA on ischaemic stroke is affected by the renal function of patients (22, 23). A recent study used renal function-normalized SUA (SUA/SCr) to reflect the endogenous uric acid levels in order to avoid the effect of kidney function, assessed the associations between SUA and stroke prognosis and showed that a lower level of SUA/SCr was associated with poor function (14). However, the effect of SUA on patients with BAD-stroke has rarely been reported. The purpose of this study was to explore the relationship between the SUA/SCr ratio and early neurological deterioration (END) in BAD stroke patients.

## 2. Materials and methods

### 2.1. Research subjects

We retrospectively analyzed the data of BAD-patients who were admitted to department of Neurology in Chengdu Second People's Hospital between January 2020 to June 2022. The inclusion criteria were as follows: (1) patients presenting within 48 h of onset; (2) patients who met the diagnostic criteria for BAD-related stroke, which was defined as follows: (1) diffusion-weighted imaging (DWI) showing that the infarct was more than three slices in the lenticulostriate artery blood supply or that the lesions extended to the surface of the pontine base; (2) computed tomography angiography (CTA)/ magnetic resonance angiography (MRA) did not demonstrate evidence of responsible vessel stenosis (>50%); and (3) various embolic mechanisms were excluded (1–3, 24). The imaging evaluation was completed 48 h after admission. The exclusion criteria included the following: (1) patients with chronic renal failure (Cr >2 mg/dL) or who required dialysis; (2) patients receiving thrombolytic therapy. At present, thrombolysis therapies possible efficacy in BAD patients remains inconclusive. A retrospective study indicated that intravenous alteplase after stroke onset reduced the incidence of END while some studies showed intravenous thrombolysis seemed to have no preventative effect on END (1, 25, 26). In order to avoid confounder bias, we chose to exclude patients receiving thrombolytic therapy. (3) patients with a contraindication to antiplatelet drugs such as various bleeding diseases or coagulation dysfunction; (4) patients with atrial fibrillation or who took coagulation medications; (5) patients with poor cardiopulmonary function or severe liver insufficiency or liver failure; (6) patients with malignant tumors; (7) patients with incomplete follow-up at 3 months post-stroke; (8) patients with a modified Rankin scale (mRS) score >1 before admission; and (9) patients with incomplete clinical data. All the patients included in this study received oral antiplatelet treatment after admission for 21 consecutive days (100 mg aspirin and 75 mg clopidogrel daily) and then were treated with long-term single antibody therapy (100 mg aspirin or 75 mg clopidogrel daily) after discharge.

### 2.2. Clinical information and assessment

The clinical data were collected by two clinicians who reviewed the electronic medical record system from our hospital. The clinical information collected included the following factors: age, sex, hypertension, diabetes, smoking, drinking, history of stroke, and history of taking antiplatelet drugs. The clinical data included the time from onset to arrival, blood pressure at admission, National Institutes of Health Stroke Scale (NIHSS) score at admission, presence of END, infarct site, and NIHSS score at discharge. END was defined as an NIHSS score increase of more than 2 points within 1 week (1, 26). All the included subjects were followed up at 3 months after the onset by telephone or face-to-face interviews to determine their mRS at 90 days. The laboratory data we collected were as follows: random blood sugar, blood lipids, urea nitrogen, creatinine, uric acid, and creatinine clearance.

The study was approved by the Medical and Health Research Ethics Committee of the Second People's Hospital of Chengdu (Chengdu, China).

### 2.3. Sample collection and assessment of the SUA/SCr ratio

We collected non-fasting blood samples for some urgent laboratory tests (including serum creatinine, serum uric acid, urea nitrogen, and random blood sugar) for all patients with acute cerebral infarction who were admitted to our hospital, and the samples were submitted for examination immediately. Total cholesterol (TC), triglycerides (TGs), low-density lipoprotein cholesterol (LDL-C), and high-density lipoprotein cholesterol (HDL-C) were tested in the fasting blood samples. All blood samples were analyzed by a Hitachi 7,600 automatic biochemistry analyzer (Hitachi, Tokyo, Japan). Laboratory physicians were responsible for reviewing the results. Renal function-normalized SUA was calculated using the SUA/SCr ratio.

### 2.4. Statistical analyses

We analyzed the data using SPSS Version 25.0 software (IBM Corp, Armonk, NY, USA). Continuous variables are expressed as the mean ± standard deviation (SD) or as the median and interquartile range (IQR). Student's *t-*test was used to compare normally distributed variables and Mann-Whitney *U* test was used to compare non-normally distributed variables. Categorical data are presented as frequencies (percentages), and the differences between the groups were compared using the chi-squared test or Fisher's exact test. The variables associated with END that had a low *p* value in the univariate analyses (*P* < 0.20) were included in the multivariate analysis. Multivariate logistic regression analysis was performed to identify risk factors associated with END. Receiver operating characteristic (ROC) analysis was used to assess the value of SUA/SCr for predicting END. Statistical significance was set at *P* < 0.05.

## 3. Results

### 3.1. Flow chart of the study

**Figure 1:**
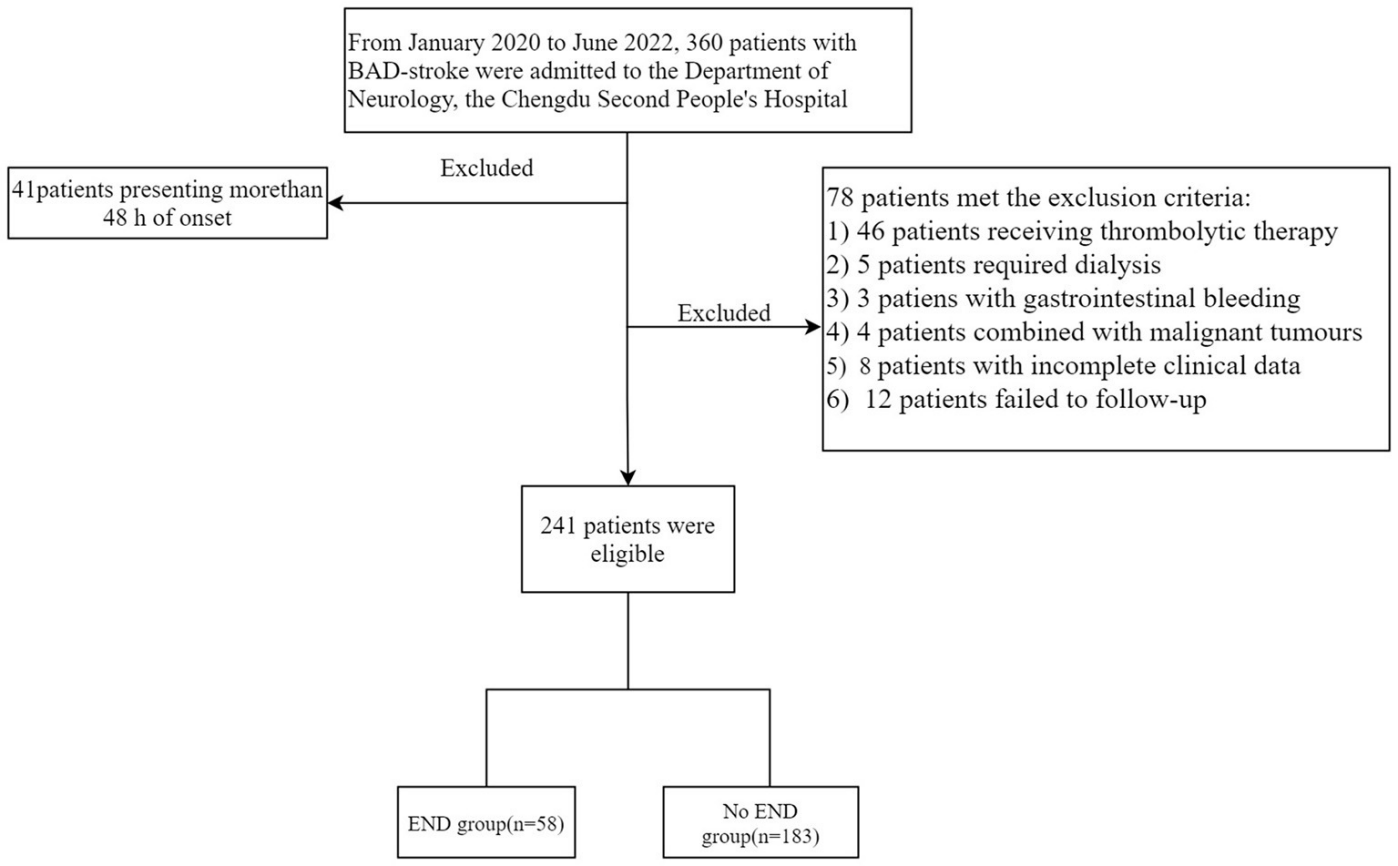
A flow chart for the patients' screening.

As shown in Figure 1, a total of 360 patients with BAD-stroke were admitted to our hospital from January 2020 to June 2022. A total of 119 patients were excluded, including 41 patients with an onset time of more than 48 h on admission, and 78 patients met the exclusion criteria (detailed information is shown in Figure 1). Finally, the remaining 241 patients were included in the final study.

### 3.2. Comparison of the clinical baseline data in the END and No-END groups

**Figure 2:**
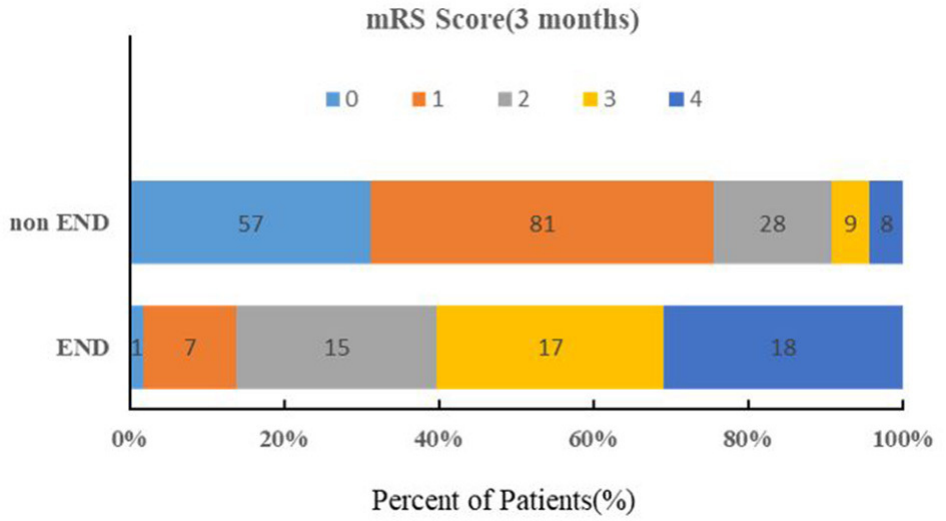
Comparison of the mRS scores at 3 months between the END group and No-END group.

The baseline characteristics of the patients are summarized in Table 1. The median age was 66.0 years, and 35.7% were female. Twenty-four percent (58/241) of all patients exhibited END after admission. In the univariate logistic regression analysis, the SUA (*P* < 0.0001), Scr (*P* <0.0001), and SUA/Scr (*P* = 0.012) in the END group were significantly lower than those in the non-END group (Table 2). There were also statistically significant differences in sex between the two groups (*P* < 0.05; Table 1). The patients who experienced END had higher NIHSS scores at discharge and mRS scores at 90 days after discharge than the patients without END (*P* < 0.0001; Table 1), and a comparison of the mRS scores between the END group and the non-END group at 3 months is shown in Figure 2.

**Table 1:** The baseline characteristics of the patients with END and the No-END group.

	**END (*n =* 58)**	**No-END (*n =* 183)**	***P***
Age, year	67.0 (57.8, 76.3)	66.0 (57.0,76.0)	0.775
Female, sex, *n* (%)	29 (50.0)	57 (31.1)	0.009
Hypertension, *n* (%)	46 (79.3)	138 (75.4)	0.542
Diabetes, *n* (%)	17 (29.3)	65 (35.5)	0.384
Hyperlipidaemia *n* (%)	15 (25.9)	61 (33.3)	0.286
Smoking, *n* (%)	15 (25.9)	53 (29.0)	0.648
Drinking, *n* (%)	5 (8.6)	30 (16.4)	0.143
History of ischaemic stroke, *n* (%)	2 (3.4)	4 (2.2)	0.595
History of taking antiplatelet drugs, *n* (%)	1 (1.7)	4 (2.2)	0.830
History of taking statin drugs, *n* (%)	1 (1.7)	4 (2.2)	0.830
**Blood pressure at admission**			
SBP, mmHg	154.1 ± 22.3	154.1 ± 21.6	0.574
DBP, mmHg	84.5 (76.0,97.0)	87.0 (78.0,97.0)	0.512
Arrival time, hours	24.0 (12.0,31.5)	24.0 (11.0,48.0)	0.998
NIHSS score at admission, hours	3.0 (2.0,4.0)	3.0 (2.0,4.0)	0.249
**Infarct site**, ***n*** **(%)**			
LSA	31 (53.4)	117 (63.9)	0.153
PPA	27 (46.6)	66 (36.1)	
NIHSS at discharge	4 (2,6)	2 (1,2)	< 0.0001
mRS at 90 days	3 (2,4)	1 (0,1)	< 0.0001

SBP, systolic blood pressure; DBP, diastolic blood pressure; NIHSS, National Institutes of Health Stroke Scale; LSA, lenticulostriate artery; PPA, paramedian pontine artery.

**Table 2:** Laboratory results in both outcome groups.

	**END (*n* = 58)**	**No-END (*n =* 183)**	***P***
Random blood sugar, mmol/L	5.8 (4.9,8.9)	5.7 (4.9,7.5)	0.839
TC, mmol/l	1.42 (1.02,1.91)	1.41 (1.02,2.21)	0.567
TG, mmol/l	4.75 (3.81,5.82)	4.73 (3.98,5.65)	0.923
HDL, mmol/l	1.16 (0.93,1.39)	1.09 (0.90,1.29)	0.296
LDL, mmol/l	2.93 (2.19,3.68)	2.78 (2.21,3.50)	0.451
SCr, mmol/l	64.0 (52.0,72.5)	73.0 (63.0,85.0)	0.000
Urea nitrogen	4.90 (4.19,5.92)	5.30 (4.30,6.30)	0.092
Creatinine clearance	77.53 (68.01,97.04)	77.02 (59.49,95.11)	0.534
SUA	258.0 (218.5,325.8)	327.0 (277.0,394.0)	0.000
SUA/Scr	4.08 (3.69,4.75)	4.61 (3.90,5.48)	0.012

TC, total cholesterol; TG, triglycerides; HDL, high-density lipoprotein; LDL, low density lipoprotein; SUA,Serum uric acid; Scr, serum creatinin.

### 3.3. Multivariate logistic regression analysis results of the factors related to END

**Figure 3:**
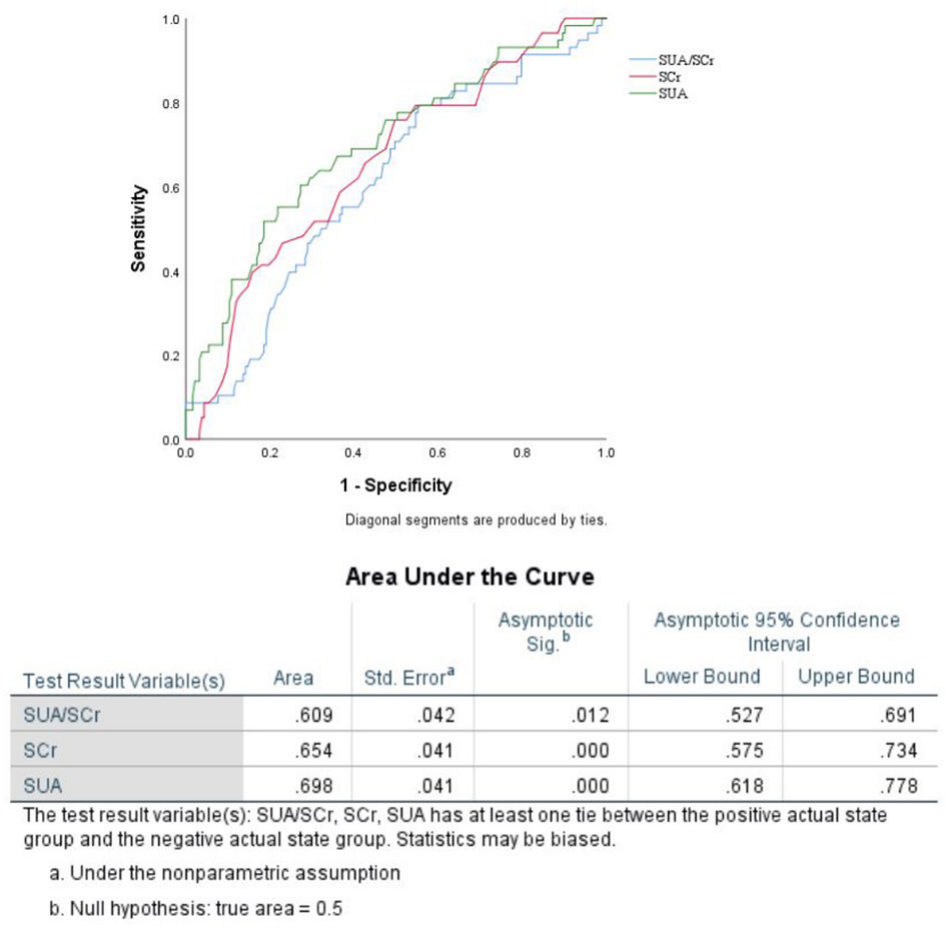
ROC analysis of SUA/SCr in predicting END in BAD patients.

**Figure 4:**
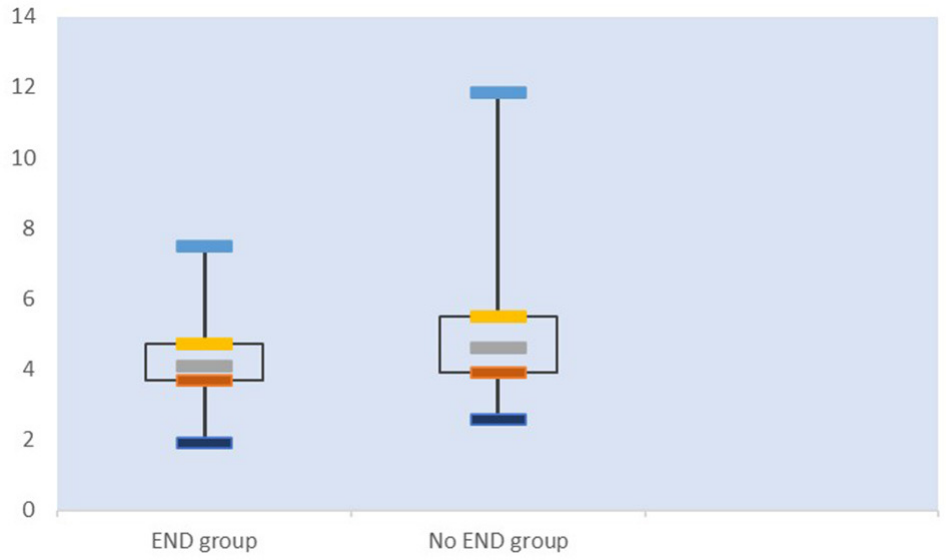
Whisker plots of SUA/SCr in the END and No-END groups.

When the factors associated with END in the univariate analyses (*P* < 0.20) were entered into the multivariate logistic regression analysis, SUA/Scr (aOR, 0.716; 95% CI, 0.538–0.952; *P* = 0.022) remained a significant factor in the final regression analysis (Table 3). The multivariate logic analysis also showed that females seemed to be more prone to END (aOR, 0.469; 95% CI, 0.245–0.898; *P* = 0.022) (Table 3). The predicted value of SUA/Scr for END was a sensitivity of 79.3%, a specificity of 44.8%, and an AUC of 0.609 (95% CI, 0.527–0.691, *P* < 0.05) (Figure 3). The optimal cut-off value was 4.76. It indicated that patients with the SUA/SCr ≤ 4.76 are more likely to experience END. Figure 4 shows the SUA/SCr levels in both groups.

**Table 3:** Multivariate logistic regression analysis results of the factors related to END.

**Risk factors**	**OR**	**95% CI**	***P***
Sex	0.469	0.245–0.898	0.022
Drinking, *n* (%)	1.441	0.497–4.174	0.501
Infarct site, *n* (%)	0.997	0.987–1.008	0.639
urea nitrogen	0.814	0.656–1.010	0.062
SUA/Scr	0.716	0.538–0.952	0.022

SUA,Serum uric acid; Scr, serum creatinine.

## 4. Discussion

The purpose of our study was to explore the association between SUA/SCr and END in BAD stroke patients. Our research found that SUA/SCr was an independent risk factor for END in BAD-stroke patients, and patients with a lower SUA/SCr were more prone to END. To the best of our knowledge, few analyses have investigated the relationship between SUA/SCr and END in BAD stroke patients.

BAD is a specific type of ischaemic stroke caused by the occlusion of the orifice of the penetrating artery (3), which is different from lacunar infarction (LI), whose pathological characteristic is fibrinoid degeneration or lipohyalinosis of penetrating artery (27). Both of them are intracranial deep brain infarction. LI was defined as an intracerebral lesion with a diameter of <15 mm and fewer than 3 slices or a lesion within the pontine parenchyma while BAD was defined as an intracerebral lesion of ≥15 mm in diameter and more than 3 slices or a lesion extending to the surface of the pontine base observed on DWI (24). zBAD stroke patients have been reported to have a tendency of neurologic worsening compared with lacunar infarction patients (2, 6). In our study, we observed that approximately 24.1% of the BAD-stroke patients experienced END, which resulted in more severe disability on discharge and higher 3-month MRS scores than the patients without END. Most of the previous studies on predictions of END in patients have focused on imaging features (8–12, 28). Studies have shown that larger infarct size or lower pons lesions may be associated with a higher probability of progressive motor deficits in patients with basilar artery branch disease (11, 28, 29). The longitudinal length of the infarcted lesion along the perforating artery was also reported to be associated with END in single subcortical infarction (10). However, there are few studies on the serum markers of END in BAD-stroke patients. Oji et al.'s small sample study showed that high mean platelet volume values on admission may be an independent biomarker for END in BAD patients (4). The effect of uric acid on END in BAD-stroke patients is currently unknown.

END after stroke has been reported to be associated with poor outcomes (30). In recent years, there have been a few studies dedicated to the relationship between uric acid and the development and prognosis of stroke. Whether uric acid is a protective or destructive factor in ischaemic stroke is still controversial. A meta-analysis by Lei et al. in 2019, which included a total of 15 high-quality studies with 12,739 acute ischaemic stroke patients, suggested that there was a significant positive association between SUA level and the outcome of ischaemic stroke (31). A retrospective study highlighted that a high SUA level (>237 mmol/L) was a protective factor for neurological functional outcome only in males and in the patients with the large-artery atherosclerosis subtype but not in females nor in the patients with other stroke subtypes (32). Chamorro et al. showed that each milligram per decilitre increase in serum uric acid was associated with a 12% increase in the odds of good clinical outcome in patients with acute ischaemic stroke (19). Wang et al.'s and Amaro's studies also support that uric acid is a protective factor for stroke (17, 20). In contrast, a meta-analysis in 2021 showed that there was no significant association between serum uric acid levels and functional outcome (21). A tertiary analysis of the URICO-ICTUS trial, which was a double-blind, placebo-controlled, phase 2b trial, suggested that uric acid therapy significantly reduced the incidence of early ischaemic worsening (EIW) compared with placebo in patients treated with alteplase within 4.5 h of AIS onset (13). As 90% of SUA is filtered and reabsorbed by the kidney, the uric acid concentration is largely influenced by kidney function. Some studies have shown that the effects of SUA on ischaemic stroke are regulated by kidney function (22, 23). A study including 3,284 AIS patients from the CATIS suggested that a high SUA concentration was a protective factor only in ischaemic stroke patients with normal renal function but not in those with abnormal renal function (22), while Falsetti et al. showed that high SUA was associated with higher in-hospital mortality for ischaemic stroke patients with kidney disease but not in patients with normal kidney function (23). Therefore, it is necessary to consider the impact of renal function when assessing the relationship between SUA and stroke. SUA/SCr has been considered to be a superior biomarker of endogenous uric acid levels (14, 16).

Our study found that BAD-stroke patients with a lower SUA/Scr had a higher risk for END and therefore poorer outcomes. A recent prospective cohort study enrolled 8,169 ischaemic stroke or transient ischaemic patients and found that a lower SUA/SCr was independently related to poor functional outcomes in patients at 3 months and 1 year after AIS. Lin et al.'s prospective observational study including 196 patients found that a BUN/Cr higher than 15 was an independent predictor of SIE (33). Then, they proceeded with a prospective interventional study that included 189 AIS patients (hydration group, *n* = 92; control group, *n* = 97), and the hydration group received an intravenous bolus (300–500 mL) of saline followed by a maintenance saline infusion, while the control group received a maintenance saline infusion. Finally, they concluded that hydration therapy may help reduce the occurrence of SIE and therefore improve prognosis (34). Our findings indicated that SUA/SCr may be a useful marker to predict END and guide the individual treatment regimen for BAD-stroke patients with high END risk. Additionally, we found that females were less likely to experience END than males.

The precise mechanism for the effect of the SUA/SCr ratio on stroke development and prognosis remains unclear. SUA is the end product of purine metabolism, and it is also well known as an endogenously generated antioxidant during hypoxia (35). The brain is a highly oxygen-demanding organ, and it is extremely dangerous for the patient when the brain suffers from oxidative stress. However, oxidative stress is one of the mechanisms that contributes to neuronal damage in AIS patients (36). It has been reported through animal experiments that uric acid, as an antioxidant, can remove free radicals and inhibit oxidative stress to protect brain tissue (35). Therefore, the antioxidant properties of uric acid may be one of the mechanisms. Therefore, we speculate that when the renal function of the two patients is the same, patients with lower levels of uric acid may be more prone to END and poor prognosis.

BAD-stroke patients are prone to END, so it is crucial to explore the factors associated with END. To the best of our knowledge, this was the first study to investigate the relationship between SUA/SCr and END in BAD stroke patients, which was a strength of our research. However, our study also has the following limitations. First, this is a retrospective study with a small sample size. Second, our study only assessed SUA/SCr at admission without continuous observations to further analyse the impact of fluctuating ratio levels. Third, we did not investigate the association between SUA/SCr and END in patients with normal and abnormal renal function separately. Fourth, Our research showed that the SUA/SCr has a good sensitivity in predicting END, but its specificity is relatively low. In daily practice, it is necessary to combine multiple factors to evaluate the risk of END. In conclusion, our study suggested that SUA/SCr was negatively associated with the risk of END in BAD-stroke patients. In clinical practice, the occurrence of END should be monitored in BAD stroke patients with a low SUA/SCr.

## Data availability statement

The original contributions presented in the study are included in the article/supplementary material, further inquiries can be directed to the corresponding authors.

## Author contributions

YL participated in the whole process of the study, including designing the study, collecting data, analyzing the data, and drafting the original manuscript. HW participated in the data collection and collation. RX and LH participated in the statistical analysis. KW and YX participated in data management, provided professional guidance in the evaluating the laboratory results, and imaging review. JW and FX designed the study and revised the manuscript. All authors have read and approved the final manuscript.
